# Maitake Pro4X has anti‐cancer activity and prevents oncogenesis in BALBc mice

**DOI:** 10.1002/cam4.744

**Published:** 2016-07-11

**Authors:** Agustina Roldan‐Deamicis, Eliana Alonso, Belén Brie, Diego Aguilera Braico, Gabriela Andrea Balogh

**Affiliations:** ^1^ Instituto de Investigaciones BiomédicasFacultad de Ciencias MedicasPontificia Universidad Católica Argentina – UCA – CONICETBuenos AiresArgentina; ^2^ Laboratorio de Hongos Comestibles y MedicinalesCentro Científico Tecnológico, CERZOS‐CONICETCamino La Carrindanga Km7Bahía Blanca‐8000, Buenos AiresArgentina

**Keywords:** Breast cancer, cancer prevention, carcinogenesis, Maitake Pro4X

## Abstract

The understanding of the molecular mechanisms of the immune tolerance induced by the tumoral microenvironment is fundamental to prevent cancer development or to treat cancer patients using immunotherapy. Actually, there are investigations about “addressed‐drugs” against cancer cells without affecting normal cells. It could be ideal to find selective and specific compounds that only recognize and destroy tumor cells without damaging the host normal cells. For thousands of years, mushrooms have been used for medicinal purposes because of their curative properties. D‐Fraction, an extract of Maitake (from the edible *Grifola frondosa* mushroom), rich in *β*‐glucans, exert notable effects in the immune system. Until now, some published articles suggest that Maitake D‐Fraction could have anti‐tumoral activity, prevent oncogenesis and metastasis in some tumor types. However, there are no clear data about Maitake D‐Fraction action on breast cancer prevention and its exact molecular mechanisms are not yet elucidated. The experiments were performed employing 25 female BALBc mice that were treated with and without Maitake D‐Fraction Pro4X or Maitake Standard for 15 days by daily intraperitoneal injection. After treatment period, all mice were implanted with murine tumor cells LM3 to induce mammary tumorigenesis. Animals were checked weekly and killed after 46 days of LM3 transplant; percentage of cancer prevention, rate of tumor growing, and overall survival were determined. Under dissection, the internal organs were evaluated histologically and genetically by RT‐PCR. We found that 5 mg/kg per day of Maitake D‐Fraction Pro4X, administered dairy during 15 days to BALBc mice was able to block more than 60% breast cancer development. However, Maitake Standard prevents oncogenesis in 26% to respect control. In this work, we found that Maitake D‐Fraction Pro4X, administered to BALBc mice, prevents breast carcinogenesis, block tumor invasiveness, reduce angiogenesis, and increase overall survival.

## Introduction

There is a great need to find new therapies for the treatment of breast cancer. While conventional therapies can extend life time in sick patients, such therapies fail to specifically target all cancer cells and thus are usually accompanied by severe side effects. An emerging new type of treatment is immunotherapy. Similar to a vaccine, immunotherapy harnesses the body's natural defense to specifically recognize and kill cancer cells. This strategy has proven to be effective in a selected patient group with certain cancers such as metastatic melanoma and renal cell carcinoma 1, 2. Unfortunately, breast cancer has not yet proven to be amenable with immunotherapy. It is unknown whether breast cancer is sometimes resistant to such forms of therapy or whether current attempts of immunotherapy are not effectively been able to boost the natural immune system. Effective treatments have still not been developed to prevent tumor cells spreading from their primary tumor, a critical step in cancer reaching different organs during metastasis.

In women with high risk of breast cancer development, drugs such as tamoxifen and raloxifene have been used to reduce their risk, but those drugs shown several side effects 3. Other drugs and dietary supplements that may help lower the risk are also being studied 4.

For thousands of years, humans have used medicinal mushrooms for their healing potential. A large folk tradition has passed on the knowledge of fungi from generation to generation, particularly in Japan and China where mushrooms are supported by decades of scientific research and are in place based firmly upon the cultural knowledge. The U.S. researchers have been exploring these fascinating organisms for their medicinal properties 5, 6. The *β*‐glucan D‐fraction of Maitake exerts profound effects on the immune system 7. It is reported that the Maitake D‐Fraction join Dectin‐1 receptors present in the outer membrane of macrophages and other white blood cells including natural killer (NK) cells and cytotoxic T (Tc) 8. These immune cells are important in the protection and fight against cancer development, since they can attack tumor cells directly. Besides increasing the ability of macrophages to engulf and destroy cancer cells, microbes, and other foreign cells, the *β*‐glucans stimulate the production of signaling proteins of the immune system, such as interleukin‐1, interleukin‐2, and cytokines 9, 10.

The *β*‐glucans has been show anticarcinogenic activity and oncogenesis and metastasis's prevention in certain cancer types. However, the exact mechanisms by which the *β*‐glucans prevent carcinogenesis are unclear.

Our previous data support the concept that Maitake D‐Fraction can influence the switching on and off of genes expressed in human breast cancer MCF‐7 cells, and thus could be able to control the breast cancer phenotype inducing apoptosis and probably involved in the reversion of the malignant phenotype 11.

Our objective here is to elucidated whether Maitake D‐Fraction Pro4X, rich in beta glucans, has inhibitory effect against mammary tumor development, and if this compound is able to reduce the metastasis and invasion through activation of genes related to tumoral phenotype inhibitory pathway.

## Materials and Methods

### Maitake D‐fraction

Maitake (D‐Fraction) Pro4X is a product commercially available from Mushroom Wisdom, Inc. from NJ. (http://www.mushroomwisdom.com). Maitake Pro4X is a purified extract containing 30% of active proteo beta‐glucan from *Grifola frondosa* mushroom. In this work, we also used a crude extract from *G. frondosa* called Maitake Standard that contains 3000 mg of mushroom extract in 120 mL. Each bottle (60 mL) of Maitake Pro4X contains 6000 mg of purified D‐Fraction, which is standardized to contain more than 1800 mg (30%) of the active proteo‐glucan. Both, Maitake D‐Fraction Pro4X or Maitake Standard contains no alcohol, sugar, yeast, mold, gluten, dairy foods, artificial colors, preservatives, or synthetic pesticides or fertilizers.

### LM3 cell line culture

The tumor cell line LM3, derived from a murine mammary adenocarcinoma in BALB/c mice, were generously provided by Dr. Elisa Bal‐Kier Joffe from Institute of Oncology, Angel H. Roffo, Buenos Aires, Argentina. Cells were maintained in MEM medium (Invitrogen, Carlsbad, CA) supplemented with 5% (v/v) FBS (Invitrogen), l‐glutamine (5 mmol/L, Invitrogen), penicillin (Invitrogen, 100 U/mL), and streptomycin (Invitrogen, 100 *μ*g/mL) at 37°C in a humidified 5% CO_2_ air atmosphere.

### Animals

In vivo experiments were carried out at the animal facility of BIOMED‐UCA‐CONICET‐Buenos Aires, Argentina. Female BALBc mice (6–8‐week‐old) weighing around 24 g were reproduced and maintained in a climate‐controlled room with a 12 h light/12 h dark cycle. Water and food were provided ad libitum. After finishing the experiments, animals were killed by cervical dislocation following the AVMA Guidelines for the Euthanasia of Animals 12.

### Experiment design

The 30 female mice (6–8 weeks old) were divided into three groups: a control group (without Maitake) containing 10 animals, Maitake Standard Group and Maitake D‐Fraction Pro4X group containing 10 mice each. The concentration used for Maitake Standard or Maitake D‐Fraction Pro4X was 5 mg/kg, administered in a final volume of 40 *μ*L diluted in PBS. The treatments were administered daily by intraperitoneal injection during 15 consecutive days. Controls were injected with PBS.

### Breast carcinogenesis induction

After treatment period, all mice were injected i.p. with 4 × 10^5^ murine tumor cells LM3 to induce mammary tumorigenesis. The mice were killed after 46 days of LM3 syngeneic transplant. Under dissection, the internal organs were evaluated. Organs of interest such as tumors, mammary gland development without visible tumor, liver, and lung were isolated. Half of breast tissue were keep in liquid nitrogen for total RNA isolation and the rest were placed in 10% formalin to performed histology studies.

### Breast tissue examination

Animals were weekly checks by visual examination and palpation of abdominal‐inguinal area to note tumor appearance. Progressively growing masses >10 mm mean diameter were regarded as tumors. The tumors were measured with a digital caliper in two perpendicular diameters and the volume was calculated as (*X* ^2^ × *Y*)/2, where *X* and *Y* represent, respectively, the smallest and largest diameter.

### Paraffin processing of tissue

Breast tissues were sectioned for preparing paraffin blocks 13. From each mouse, the tissues were drop‐fixed in a 10% formalin solution for a minimum 48 h at room temperature. After 48 h of fixation, tissues were moved into 70% ethanol. Breast tissues were dehydrated through a series of growing graded ethanol baths, cleared in xylene and embedded in paraffin. Finally, tissues were sectioned in 10 *μ*m slides using a microtome.

### Hematoxylin and eosin stain

The tissue architecture and ultrastructural details were preserved and stained with hematoxylin–eosin or periodic acid‐Schiff staining modifications in paraffin sections 14.

### Angiogenic index

In order to determine density of blood vessels in the paraffin blocks from mammary tissues, we did employ the published methodology 15. The measure was in blood vessels average/mm^2^.

### Total RNA isolation from mammary tissue

In this study, we employed frozen breast tissue (tumoral or normal) from each animal. The RNA was isolated by duplicate using Trizol (Invitrogen, Inc.) following the classic phenol purification method 16. The concentration and the quality of total isolated RNA were measured in spectrophotometer considering a ratio 260/280.

### RNA purification

In order to purify the total RNA isolated from each breast tissue, we utilized the QIAamp RNA Mini Kit from QIAGEN, Valencia, CA, following the author instructions. Briefly adding 700 *μ*L of Buffer TW1 to the sample, centrifuged at 8000*g* during 15 sec at room temperature, discarded the flow trough, change the tube and washed RNA adding 500 *μ*L of Buffer RPE twice, centrifuged at 8000*g* during 15 sec at room temperature, discarded the flow trough. A new microtube was placed and 30 *μ*L of free RNases water was added, and centrifuged at 10,000*g* for 1 min. The eluted RNA was collected and its concentration was measured using spectrophotometer at 260 nm, and before amplification by RT‐PCR, an 1% agarose gel was run to visualize its quality.

### Reverse transcription (RT) reaction

The RT reaction contained 1 *μ*g of purified total RNA, 5 *μ*L of Buffer M‐MLV 5X (Promega, Madison, WI), 10 mmol/L each of dATP, dGTP, dCTP, dTTP, 0.5 mg/mL OligodT primer, and 1 *μ*L of M‐MLV RT enzyme (Promega). A 10 *μ*L mixture of total RNA and primer was heated for 5 min at 70°C, cooled on ice at least 2 min, and quickly spun before adding the remaining components (final volume 20 *μ*L). The reaction proceeded for 1 h at 40°C and was terminated by heat inactivation at 70°C for 15 min. RT products were stored at −20°C until used.

### Polymerase chain (PCR) reaction

The nucleotide sequence of each primer used in this work is indicated in Table 1. Each reaction (PCR) was performed following the established Protocols 17, briefly using 50 ng of cDNA, 1X PCR Buffer with 1.5 mmol/L MgCl_2_ (Amersham Biosciences, Piscataway, NJ), 200 *μ*mol/L dNTPs (Amersham Biosciences), 10 *μ*mol/L of each primer (see Table 1), and 1 U Taq DNA polymerase (Amersham Biosciences) in a final volume of 12.5 *μ*L. The PCR protocol was the same for each amplified gene region except for the annealing temperature which was specified by each primer pair. The amplification conditions were: 5 min initial denaturalization at 94°C, 35 cycles of 30 sec at 94°C, 60 sec at the annealing temperature (annealing) of each pair of primers and 30 sec elongation at 72°C. Followed by a final extension of 10 min at 72°C and maintained at 4°C until removal from the thermocycler samples.

**Table 1:** Nucleotide sequence of forward and reverse primers to amplify ABCG2, CUL3, IGFBP5, PTEN, and SPARC genes by RT‐PCR method

Primer	Nucleotide sequence 5′→3′	*T*_m_ (°C)	Size of fragment (bp)
*β*‐Actin forward primer	GGATGCAGAAGGAGATCACTG	60.0	90
*β*‐Actin reverse primer	CGATCCACACGGAGTACTTG		
ABCG2 forward primer	GCTGTGGAGCTGTTCGTAGT	61.8	664
ABCG2 reverse primer	AGTCCGTTAAAGGGGGAAATTAAGA		
CUL3 forward primer	TCCCCAGGTCTTCAGTGTTGA	62.5	884
CUL3 reverse primer	TTGGAAGCACAGAGGAACGG		
IGFBP5 forward primer	CAGTATACCCATCACCCCGC	63.4	989
IGFBP5 reverse primer	ACAGCTGACCTCCTCCGTAT		
PTEN forward primer	GTGGTCTGCCAGCTAAAGGT	62.4	1000
PTEN reverse primer	AAGTGCAAAGGGGTAGGACG		
SPARC forward primer	TCTGGGTAGCACACAGCCTA	62.4	951
SPARC reverse primer	TCTCAAAGTCTCGGGCCAAC		

β‐Actin amplification was used as control.

### PCR products visualization and semiquantification

To visualize the amplified RNAs, samples were run in 3% Agarose gel at 120 V during 40 min and stained with ethidium bromide and visualized in Benchtop 2UV transiluminator (Bio DOC‐It Imaging System; UVP, LLC Ultra‐Violet Products Ltd, Upland, CA). Semiquantification was done using the densitometric analysis using NIH Image (http://rsb.info.nih.gov/nih-image).

### Statistics analysis

The effect of treatment was tested by ANOVA 18. Differences among means were assessed using Student's *t*‐tests. Experiments were done by triplicate as independent experiments. Variability was expressed as SE. Differences were considered significant at *P* < 0.05. Kaplan–Meier survival curves and the log‐rank test were used to analyze the differences between the two groups (Maitake Standard and Maitake Pro4X) with respect to control.

## Results

### Effect of Maitake D‐fraction Pro4X in the breast cancer prevention

**Figure 1:**
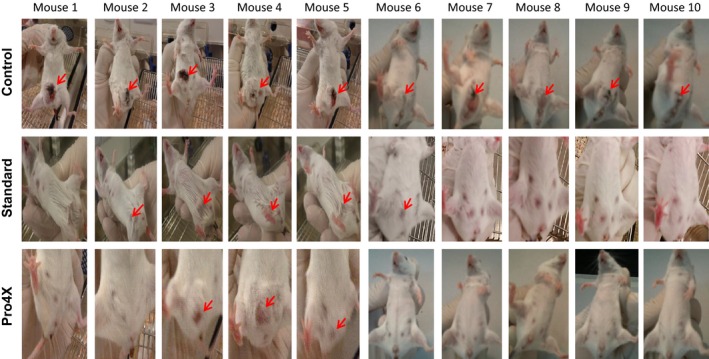
Photographs of the abdominal region of 30 BALBc mice in the Control, Maitake Standard, and Maitake Pro4X groups. After 15 days of treatment with each compound in a concentration of 5 mg/kg, breast tumorigenesis was implanted in mice peritoneal region as indicated in Methodology and breast tumorigenesis were checked by weekly. Red arrows indicate the breast tumor in each mouse. *P < 0.01.

In order to demonstrate if the purified extract Maitake D‐Fraction Pro4X has an effect in the breast cancer prevention; three independent experiments were done employing female nulliparous Balbc mice. Figure 1 shows a representative picture of mice abdominal area from each condition after 30 days of tumor challenge. From this experiment, we observed 100% of breast tumorigenesis (10 out 10 animals) in the control group. Five out 10 animals treated with 5 mg/kg of Maitake Standard (Standard) developed breast tumor. However, only three out 10 mice developed mammary tumors in the condition treated with purified extract Maitake D‐Fraction Pro4X (Pro4X) (Fig. 1).

**Figure 2:**
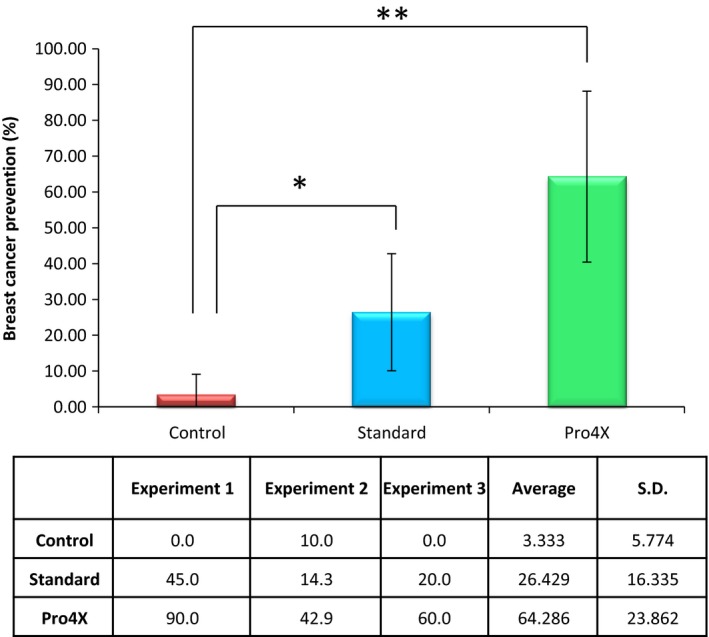
Breast cancer prevention induces by Maitake. This figure indicates the average results obtained from three independent experiments. *P < 0.05, **P < 0.01.

The average from three independent experiments of prevention against breast tumorigenesis development in animals from Control group was 3.333 ± 5.774 (Fig. 2), which was significantly different from the prevention generated by Pro4X (64.286 ± 23.862, *P* < 0.01) and also were different from those results obtained from employing Standard (26.429 ± 16.335, *P* < 0.05) as well (Fig. 2).

### Effect of Maitake D‐fraction Pro4X in the tumor grows that escape to treatment

**Figure 3:**
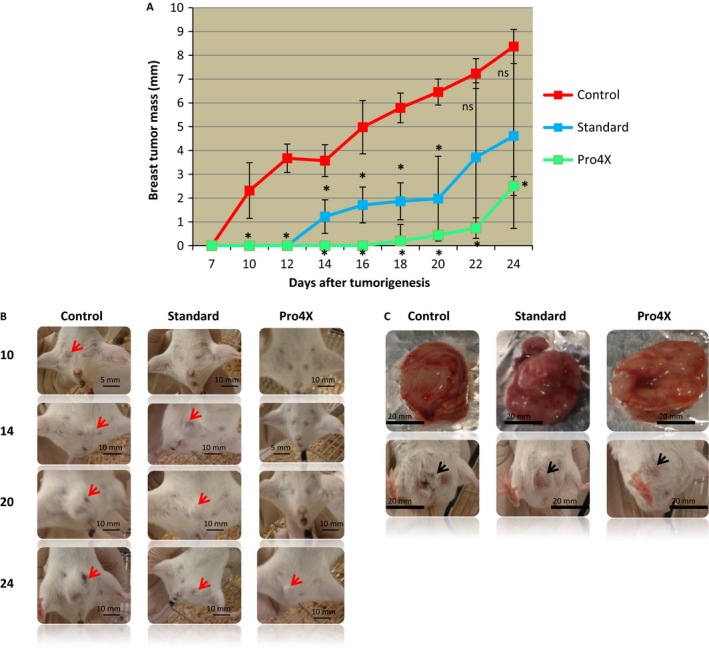
Breast tumorigenesis grows. (A) Growing curves from 7–24 days after tumor bearing in mice treated and untreated with Maitake Standard or Pro4X, respectively. (B) The abdominal area of representative's mice from each condition at 10, 14, 20, and 24 days after tumorigenesis initiation. In the pictures, the red arrows indicate the presence of breast tumors. (C) Pictures of breast tumor from a representative mice from each group. In this figure, black arrows indicate the location of breast tumors *P < 0.01.

After analyzing the prevention percentage in each treatment, it is important to study the tumor growth curve that did not respond to Maitake treatment and escaped its control. From Figures 1, four out 10 and three out 10 animals escaped the Maitake Standard and Pro4X prevention, respectively and developed breast tumors. If the tumor area is comparable between groups, we are looking for to study even those events. Figure 3A indicated the mammary tumor growing curve (longitudinal size in cm) in each mouse with breast tumors from each group since 7–24 days after tumor challenge. From the graphic, it was observed that breast tumor in the animals from control group grew linearly since 10–24 days after tumor challenge; however, the breast tumorigenesis after Pro4X treatment did grow slowly at the same time and at 24 days, achieved a similar size compare to the Standard treatment (See Fig. 3A). In the Standard treatment, the tumor grows curve was significantly different (*P < 0.05) to compare with Control until day 20; however, after 22 days, the tumor grew in a similar size as the group without Maitake (Fig. 3A). At 46 days, after tumor challenge (the end of experiment), the tumor area (cm^2^) did not achieve any significant difference between groups.

From another side, it was found macroscopic differences in the breast tumor between groups. At 46 days after tumorigenesis, challenge was observed that breast tumors without Maitake (control) were solid and with irregular edges; however, tumors from Maitake Pro4X were same size than controls but full of liquid, not solid, with net tumor round edges (Fig. 3C). Tumors from Maitake Standard treatment showed a macroscopic aspect similar to control.

### Effect of Maitake D‐fraction Pro4X in the histology of breast cancer developed

**Figure 4:**
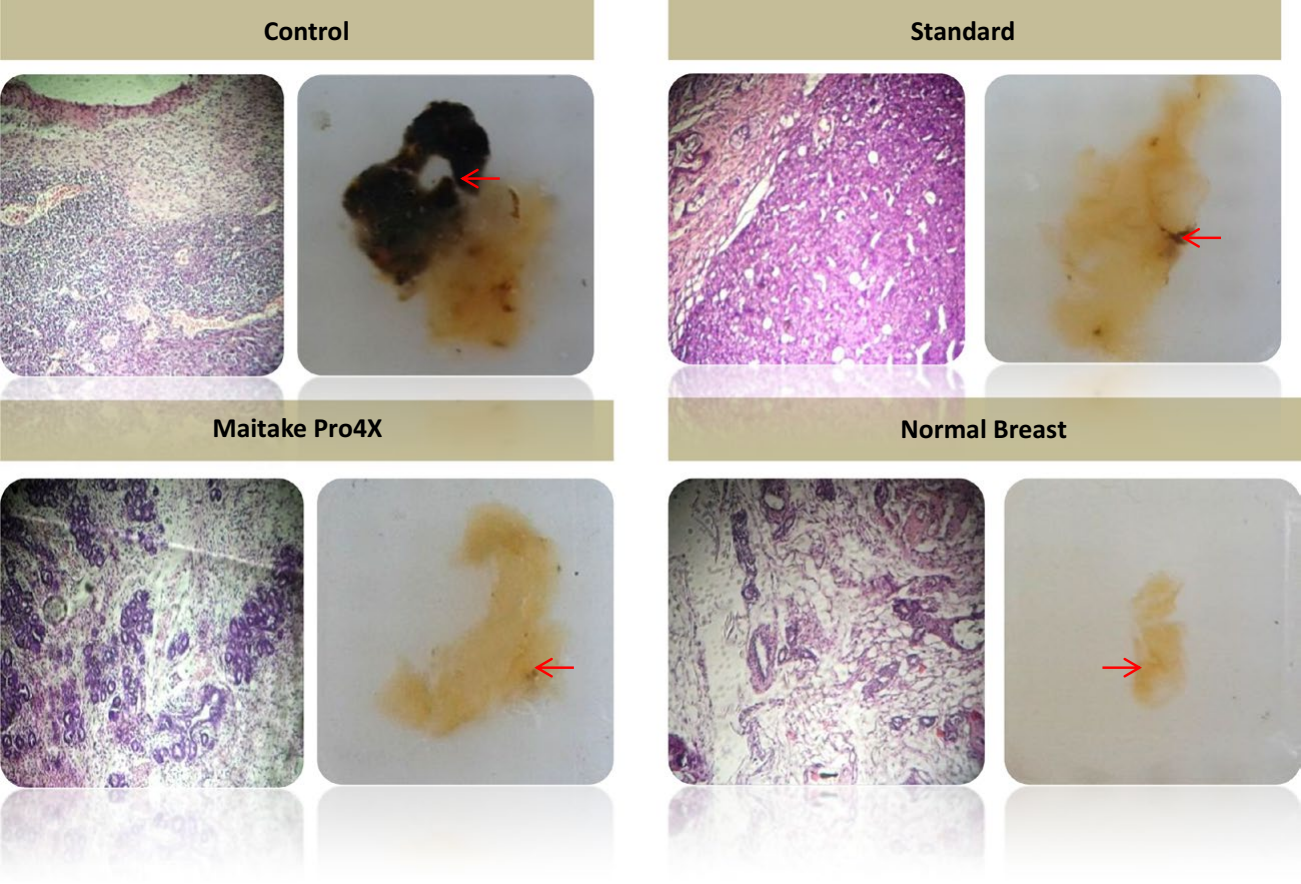
Histology of mammary tissues from each treatment group with or without Maitake. The picture indicated as normal breast corresponded to breast tissues without tumor developed after Maitake Pro4X treatment. Right picture shows the paraffin block of breast tissues, the arrow indicate the amplified region and left picture shows the corresponding microscopy breast histology.

The differences found in the macroscopic aspect of tumors treated with or without Maitake address us to perform histology studies from mammary tissue, paraffin blocks were performed, 5 mm slides were stained with H&E and observed with optical microscope. The histology slides of mammary tissues from each treatment group with and without Maitake are illustrated in Figure 4. The last figure, Normal breast represents the breast tissues without tumors developed after Maitake Pro4X treatment. The histology studies from mammary tumors indicated that breast tumors from Control group corresponded to hyperplasic and poorly differentiated breast tissue comparable with invasive cancer. Breast tumors treated with Maitake Standard were found similar to control group; however, mammary tumors treated with Maitake Pro4X were histologically differentiated from other groups, were well differentiated, not invasive with net borders, and less number of cells comparable to a benign breast tumor (Fig. 4). These surprising results address us to think that Maitake Pro4X could be avoiding the metastasis process in organs such as lung or liver. We next evaluated metastasis.

### Effect of Maitake Pro4X on tumor necrosis

**Figure 5:**
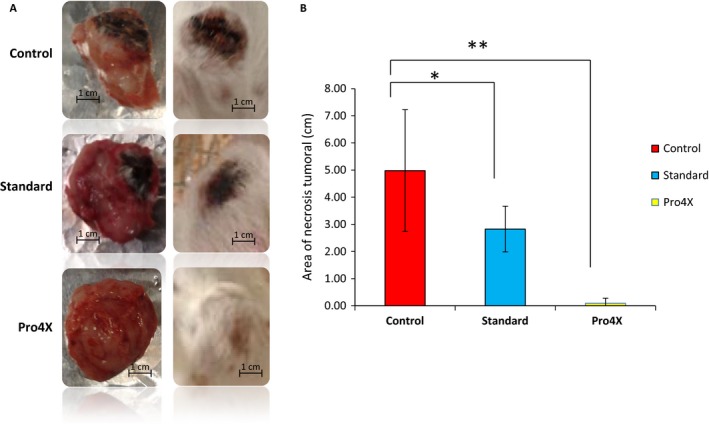
Maitake Pro4X treatment reduces tumor necrosis. In (A), represents the pictures of breast tumors isolated (left) and in vivo (right) in all groups. (B) represents the area of tumoral necrosis (cm) measured in each group of mice. *P < 0.05, and **P < 0.01.

Another aspect that is important to analyze in this work is the appearance of necrosis in the developed breast tumors. Figure 5A shows the macroscopic aspect of breast tumors with and without Maitake at 46 days after tumor challenge. After measuring the necrosis area (cm) from breast tumor in each animal group (Fig. 5B), it can be concluded that control developed breast tumors with higher necrosis area compared to Maitake Standard (*P < 0.05) or Maitake Pro4X (*P < 0.01). The treatment with Pro4X practically did not developed necrosis in their tumors (Fig. 5).

### Effect of Maitake Pro4X on metastasis in liver and lung tissues

In order to verify the presence or absence of metastasis, lung and liver were isolated from each tumor‐bearing mice treated with or without Maitake Standard or Maitake Pro4X. Weigh, macroscopic aspect, and size of lung and liver from those mice were checked.

**Figure 6:**
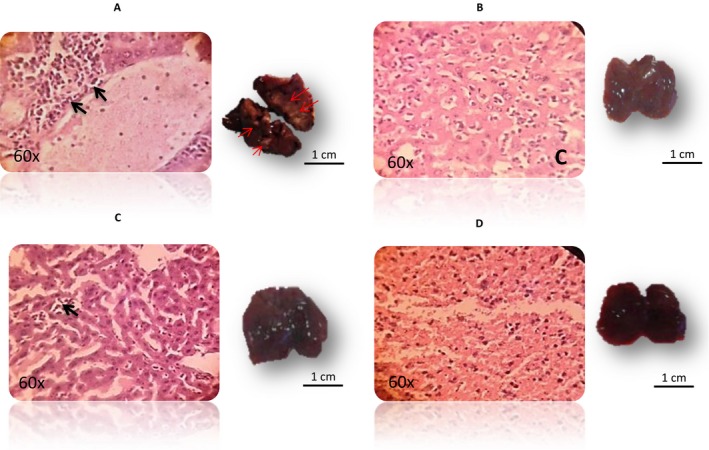
(A–D) The liver tissues histology (left) of tumor‐bearing mice from Control, Maitake Pro4X, Maitake Standard, and the normal liver from animals without breast tumor that resist carcinogenesis after treatment with Maitake Pro4X, respectively. Black arrows represent the cell proliferation area. Right pictures from each histology, represents the liver tissue in each condition. Red arrows represent the metastasis area.

The lung and liver average area (cm^2^) from each experimental group were analyzed. No significant differences in sized of lung or liver tissues from all animals in each experimental group were found (data not shown). But nevertheless, macroscopically, liver tissues from Control Group were completely different from those treated with Maitake (Fig. 6A). The mice's liver without Maitake treatment were colorless and rigid, with signs of metastasis (indicated with red arrow in Fig. 6A right). Liver tissue histology from tumor‐bearing mouse in the control group showed and confirmed cell proliferation and the hyperplasia (Fig. 6A left). However, liver tissues from Maitake Standard (Fig. 6C) or Pro4X (Fig. 6B) treated were darker, with texture and aspect normal. In the Standard treatment, histology was observed in few regions of the liver tissue with hyperplasia and proliferation that was not significant compared to control. Figure 6D shows a normal liver tissue from animals without breast tumor that resist carcinogenesis after Pro4X treatment. The histology studies from liver tissues indicated that the mice from control group have a liver tissue with bigger blood vessels, with liver structure different than normal and some mitotic changes. However, the liver tissues treated with Maitake Pro4X were not found different than normal liver tissue.

**Figure 7:**
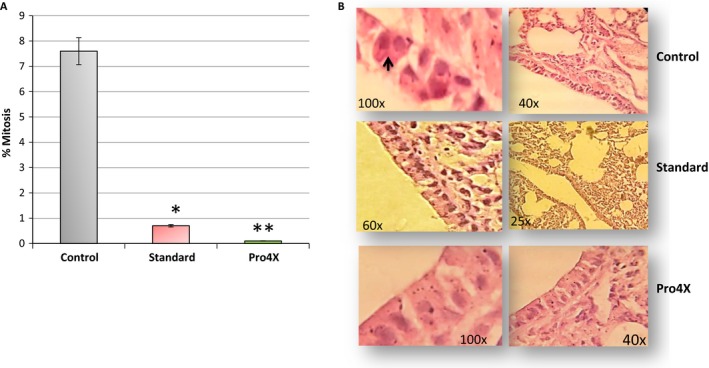
Study of mitogenesis in lung tissues. In (A) represents the % of mitosis in lung tissue at each condition. *P < 0.05, and **P < 0.01. (B) The pictures from the histology of lung tissue in every group: Control, Standard and Pro4X. The right pictures represent a magnification of left image at 60–100X. Black arrow in (B) from control group represents the mitosis.

In the macroscopic studies of lung tissues, we did not observe morphological differences between tumor‐bearing mice and non‐tumor‐bearing mice. However, surprisingly, were observed higher mitosis percentage in the lung histology sections from animals in the control group (7.50 ± 0.7) compared to Maitake treatments (0.8 ± 0.1, *P* < 0.05 in the Standard and 0.1 ± 0.02, *P* < 0.001 in the Pro4X) (Fig. 7A). Black arrow indicated in Figure 7B shows the mitosis in the bronchiolar area from lung tissue corresponding to each group of treatment. No statically differences were observed between percent of mitosis in both Maitake treatments compared to normal lung tissue.

### Effect of Maitake extract in the angiogenesis process

**Figure 8:**
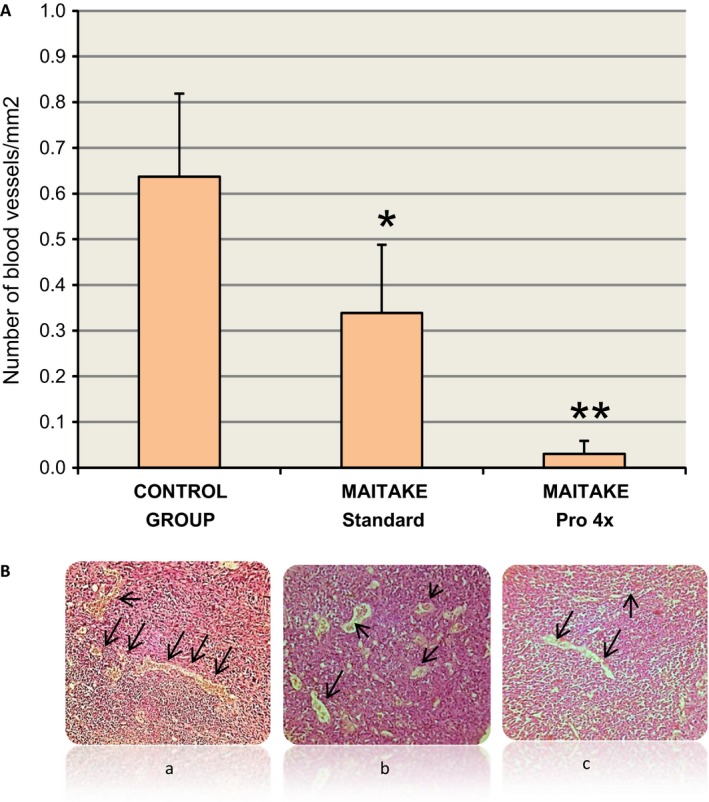
(A) The graphics of average of blood vessels density/mm^2^ in each group. (B) shows the pictures of breast tumors analyzed, Black arrows indicated the size of blood vessels in each condition. *P < 0.05, **P < 0.01.

The Angiogenic index in the tumoral breast tissues was measured in order to establish if Maitake D‐Fraction Pro4X are able to exert an antiangiogenic effect. Figure 8A shows the graphics of average of blood vessels density in each group. Figure 8B shows the pictures of breast tumors from the treatment groups a, control; b, Standard and c, Pro4X, respectively. From Figure 8, it was observe that the blood vessels number/mm^2^ in breast tumor tissues from Control Group mice (0.637 ± 0.182) were significantly higher (*P* < 0.05) than breast tumors treated with Maitake Standard (0.339 ± 0.149) or than Maitake Pro4X treatment (0.031 ± 0.028). Figure 8B shows the slide sections from breast tumors analyzed. Black arrows indicated the size and location of blood vessels in each tissue.

### Effect of Maitake extract in the relative overall survival in BALBc mice

**Figure 9:**
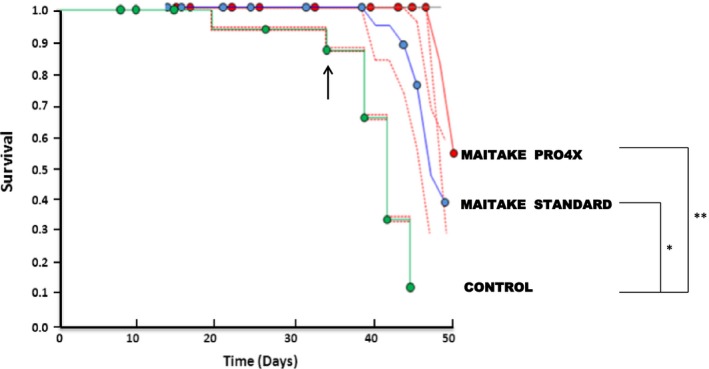
Kaplan–Meier overall survival curves. The graphic represents of overall relative survival from 7 to 50 days after tumorigenesis initiation. A green line represents the control group, a blue line represents the Standard treatment, and a red line indicates the survival after Maitake Pro4X treatment. *P < 0.05, **P < 0.01.

The overall relative survival in the mice from each group of treatment were analyzed using Kaplan–Meier curves since the beginning to the end of the experiment at 46 days after tumorigenesis initiation (Fig. 9). From those curves, it was observed that since 35 days to the end, the overall survival were significantly different between Maitake treatments (1.00 ± 0.00, both Standard and Pro4x) compared to control group (0.87 ± 0.086). From this time, survival decreases more rapidly in control compared to Standard. At the end, at 46 days after tumor challenge, the overall survival is reduced to 0.108 ± 0.099 in Control, which was significantly different compared to Standard (0.82 ± 0.12, *P* < 0.05) or Pro4X (1.00 ± 0.00, *P* < 0.01) (Fig. 9). Overall survival in Pro4X decreases to half at 50 days after tumor challenge.

### Effect of Maitake PRO4X on specific gene expression related to tumoral phenotype inhibition

**Figure 10:**
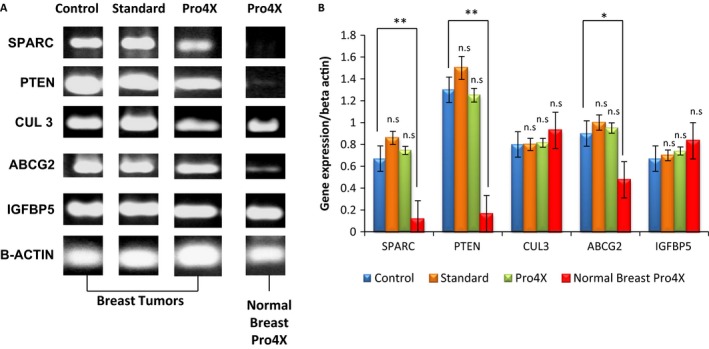
Gene Expression analysis. (A) The gene expression at mRNA level in all the conditions. (B) Relative quantification of each RT‐PCR reaction. *P < 0.05, and **P < 0.01.

With the objective to determine if Maitake D‐Fraction PRO4X modifies the gene expression of tumoral phenotype, total RNA was isolated from all breast tumors of experimental groups and from the mammary glands of non‐tumor‐bearing mice from Maitake Pro4X treatment. ABCG2, CUL3, IGFBP5, PTEN, and SPACR gene expressions were modified after Maitake treatment in MCF‐7 cells as were previously published by us 11. Figure 10A shows the RT‐PCR bands in the agarose gels from each of those genes in breast tumors after treatment with or without Maitake. The last column represents the breast tissues treated with Maitake Pro4X without tumor indicated as Normal breast Pro4X in the Figure 10A. Beta actin gene expression was used as control. This study showed that SPARC gene was differentially expressed in all the tumor conditions. SPARC gene expression was up‐modulated in the breast tumor tissues treated or untreated (control, 33.5 ± 0.70) with Maitake Standard (43.0 ± 0.90) or Pro4X (37.25 ± 0.70) (Fig. 10B). A clear down‐modulation (5.0 ± 0.01, *P* < 0.01) was observed in SPARC gene expression from normal breast tissues after Pro4X treatments that were resistant to carcinogenesis.

With respect the gene expression of PTEN, similar level of mRNA was observed for control (65.00 ± 1.30), Standard (75.0 ± 1.50), or Pro4X (62.5 ± 1.30) in breast tumors (Fig. 10A and B). However, PTEN gene expression was significantly down‐modulated in normal breast tissues treated with Pro4X (7.0 ± 0.20, *P* < 0.01).

ABCGs gene are also expressed differentially in breast tumors compared to normal breast tissue after Pro4X treatment. ABCGs gene expression were not significantly different in control (45 ± 0.90), compared to Maitake treatment (Standard, 50.0 ± 1.00 or Pro4X, 47.5 ± 1.00) in breast tumors. However, a significant down‐modulation was observed (20 ± 0.50, *P* < 0.05) in normal breast tissues resistant to carcinogenesis after Pro4X treatment (Fig. 10A and B).

Moreover, CUL3 and IGFBP5 gene expression analysis did not show a significant difference between tumors or normal tissues. CUL3 and IGFBP5 genes were expressed in all conditions at same level (Fig. 10A and B).

## Discussion

The antitumor properties of Maitake D Fraction are attributed to its immunomodulatory effect. However, a direct effect of Maitake on prostate cells 19, stomach 20, kidney 16 and breast cancer 21 by inducing apoptosis has also been reported.

The potential effects of various natural compounds such as herbs and dietary supplements are investigated to reduce the risk of cancer. It has been reported that *β*‐glucans of Maitake possess the ability to inhibit tumor growth administered either orally or intraperitoneally 22. In this work, the effects of a purified extract of Maitake D‐Fraction and Pro4X were investigated in comparison with the crude extract of *G. frondosa*, edible mushroom called Maitake Standard in the prevention of mammary tumorigenesis in BALBc mice. Therefore, they are essential in the future to isolate and chemically characterize (determine the composition and structure) the active components of both Maitake Standard and D‐Fraction Pro4X.

The results suggest that 5.0 mg/kg of Maitake D‐Fraction Pro4X prevents mammary tumorigenesis in more than 64% in BALBc mice by intraperitoneal injection, compared to Maitake Standard prevention that achieve an average of 26.43%. Concluding from those results that Maitake Pro4X has a significant effect on the prevention of breast carcinogenesis compared to Maitake Standard. Similar results were obtained by Nanba and Kubo 23, whom treated BALBc mice with 0.2 mg of Maitake D Fraction for 15 consecutive days, and after tumorigenesis induction with carcinogen 3‐methylcholanthrene, they obtained about 69% inhibition of hepatic carcinogenesis (compared with 7% in the control group).

Those results can be explained considering that Maitake Standard product is a nonpurified D fraction, and may contain other components than 1,3 or 1,6 beta glucans, the known active compounds responsible for the therapeutic actions in breast cancer. Studies to date have identified a number of compounds and elucidated underlying mechanism. However, research is needed to elucidate the different roles of multiple active compounds and the pathways involved.

In addition to preventing mouse breast carcinogenesis, Maitake Pro4X significantly reduces tumor necrosis and aggressiveness compared to untreated condition, suggesting that this compound is also involved in the reduction of invasiveness and metastasis. The inhibition of metastasis is the key for delaying cancer progression and prolonging patient's survival. In previous studies, using mice, several authors have reported the ability of Maitake D‐Fraction to prevent the liver 24 and lung 8 metastases development. It has been suggested that the mechanism involved the activation of the immune system, which facilitates the elimination of tumor cells in the blood or lymphatic circulation. In this work, the liver and lung tissues from Maitake‐treated and ‐untreated mice were checked to verify the presence or absence of metastases in these organs. Concerning to liver tissue, differences between groups were observed. In the control group, dilated vessels and liver tissue with abnormal structures were observed. Instead, the treated groups with Maitake Standard or Pro4X did not show morphological differences compared to normal tissues. We observed that maitake treatment significantly reduce the cell proliferation and percentage of mitosis in lung tissue compared to untreated condition. However, further studies are needed to determine with certainty whether histological abnormalities of the control animals are due to metastasis.

Angiogenesis is a fundamental process in tumor growth and metastasis generation. The results obtained in this work suggest that density of blood vessel was significantly lower in treated groups with both Maitake Standard and Pro4X with respect control group; moreover, the lowest blood vessel density corresponded to Maitake Pro4X‐treated group. These results are in agreement with a previous study which showed that an aqueous extract of Maitake inhibited the in vivo or in vitro VEGF induced angiogenesis both in vitro and in vivo 25. Regarding the histology of the mammary tissues, it is important to note that mammary tumors treated with Maitake Pro4X have decreased proliferation and increased differentiation compared to control group. This is consistent with the idea that Maitake D Fraction could help to reduce the process of mammary carcinogenesis and tumor progression.

Despite advances in the treatment of breast cancer, there are still many obstacles to overcome. In many cases, the treatment is ineffective due to resistance of tumor treatment or due to the presence of metastases. Moreover, existing therapies can produce adverse side effects due to their specificity. For these reasons, new research and carefully look more lenient to treat cancer patients are need it, inhibiting metastasis, increasing survival and improving life quality. Our results shown that 5 mg/kg of Maitake treatment increases significantly the overall survival from 10% in the control to more than 75% at 46 days after tumor challenge. Extrapolating with human life time, 46 days represents more than 10 years in human, meaning that, this natural and purified compound could potentially increase the overall survival of more than 10 years in untreated patients with high grade of breast cancer.

Later studies were performed to determine whether the administration of Maitake Pro4X‐modulated gene expression on breast tumor tissue. ABCG2, Cul3, IGFBPR5, PTEN, and SPARC genes levels of expression were related to the malignant phenotype in tumor cells, and those differential expressions were found to be modulated by Maitake in our previous studies 11.

One of the genes analyzed was secreted protein, acidic, cysteine‐rich (osteonectin) (SPARC), whose role in mammary carcinogenesis and metastasis is controversial. Some studies have linked high basal expression of SPARC with a poor prognosis and decreased survival in breast cancer patients with HER2+ 26, invasive ductal carcinoma 27, and a shorter time to recurrence in patients with ductal carcinoma in situ 28. It has also reported the absence of SPARC expression in benign tumors 27. On the other hand, SPARC have been associated with low expression and decreased survival in poor prognosis patients with luminal subtypes A or triple negative breast tumors 29. It has also been reported that its endogenous up‐modulated expression in cells of breast cancer MDA‐231 cells are related to reduce metastases and reduce invasiveness activity 30. In this work, it was found that SPARC gene is little or not expressed in the normal breast tissue resistant to breast carcinogenesis, from mice treated with Maitake Pro4X, whereas SPARC was highly expressed in breast tumor tissues. These results are in agreement with the work of Watkins et al. 31 who reported higher levels of SPARC gene in breast tumor tissue compared to normal breast tissue. Suggesting that Maitake D‐Fraction Pro4X could contribute to maintain the down‐modulated SPARC gene expression to prevent breast carcinogenesis. However, when the SPARC inhibition threshold induced by Maitake is overcome, breast cancer will be developed without SPARC control. Moreover, we can hypothesize here that Maitake D‐Fraction Pro4X could induce prevention against breast carcinogenesis through down‐modulation of mRNA SPARC gene expression level.

Phosphatase and tensin homolog (PTEN) was reported as a tumor suppressor gene that negatively regulates oncogenic pathway of phosphatidylinositol‐3‐kinase (PI3K)/Akt 32, was also found mutated in various cancers and its expression has been associated with tumor progression in a dose‐dependent fashion 33. In this work, it was found that PTEN gene was not expressed in normal breast tissue corresponding to a breast carcinogens‐resistant tissue. Moreover, here, it was found that PTEN gene was overexpressed in breast tumor tissues, treated or untreated with Maitake. This data could suggest that probably PTEN is shown up‐modulated due to a mutation and its accumulation in breast cancer tissue, however, in the normal breast tissue, the tumor suppressor PTEN are not expressed or is down‐modulated due to nonmutation and nonaccumulation. These results suggest that the PTEN promoter gene variation in expression could have an effect on increased metastatic potential and progression of the tumor. Concluding that Maitake Pro4X could regulate the expression of the PTEN gene involved in breast carcinogenesis. However, further studies will be needed to determine the exact role of functional PTEN variant proteins.

Cullin 3 (Cul3) gene expressions was also studied in breast tumors from Maitake‐treated and ‐untreated mice. Cul3 plays an important role in protein degradation by the proteasome 34. Cul3 loss expression has been detected in a wide range of human liver cancers and correlated directly with tumor de‐differentiation 35. Recently, Cul3 gene expression level was correlated with tumor stage in breast tissues 34. Also, Cul3 has been involved in the ubiquitination of breast cancer metastasis suppressor 1 gene (BRMS1), which inhibits metastasis 36. In this study, no differences in Cul3 gene expression in mammary tissues with or without tumor, Maitake treated or untreated were observed; so that it cannot be associated with the expression level in the tumorigenesis process.

ABCG2 is involved in multidrug resistance in breast tumors 37, 38, 39, 40. Specifically carries different chemotherapeutic agents and is involved in the development of multiple drug resistance in cancer cells. Increased ABCG2 gene expression has been reported in Mitoxantrone‐resistant breast cancer cell line with respect to a sensitive Mitoxantrone MCF‐7 cell line 40. The results in this work indicate that ABCG2 gene is not expressed or is slightly expressed in normal breast tissue resistant to carcinogenesis induced by Maitake, but is expressed in breast tumors treated or untreated with Maitake, suggesting probably that ABCG2 gene expression could have a complementary role involved the oncogenesis de‐differentiation, meaning that when is over‐expressed, increases the tumorigenic potential of transformed cells, and the resistance to multidrugs, as occuring in the breast tumors treated or untreated with Maitake. However, when ABCG2 is slightly expressed or not expressed, directly or indirectly inhibited the oncogenesis and reduce the tumorigenic potential of tumoral cells, and maintain the threshold of tumorigenesis low, or inhibited as happen with the breast tissue without tumor which was resistant to carcinogenesis after treatment of Maitake Pro4X. The question is why Maitake Pro4X did not protect 100% of animals against breast cancer development? To answer this question, we need to continue to investigate whether the oncogenesis protection mediated by Maitake Pro4X is related to the environmental exposure and the level of effectors’ immune response in each individual.

Finally, the expression of IGFBP5 gene was studied. It has been shown that IGFBP5 prevents cell growth of human breast cancer either in vitro or in vivo, causing G2/M cell cycle arrest and induction of apoptosis associated with increased mRNA expression of pro‐apoptotic BAX, and decreased expression of antiapoptotic BCL‐2 41. In an in vitro assay, a reduction of IGFBP5 gene expression in human breast cancer MCF7 Tamoxifen‐resistant cells was also observed 42. In this work, no difference was observed in the IGFBP5 gene expression between tumor and normal breast tissue treated or untreated with Maitake, so it can not establish a relationship between Maitake treatment and the expression of this gene and development of breast tumorigenesis in vivo. No differences between tumors from Maitake treated and untreated were observed; so, in conclusion, Maitake D‐Fraction did not exert an effect on the expression of IGFBP5 gene in breast tumorigenesis in BALBc mice model.

In conclusion, in this work, we demonstrated that intraperitoneal administration of 5 mg/kg Maitake D‐Fraction Pro4X during 15 days prevents breast tumorigenesis in more than 60%, increases the overall survival from 10 to 75%, reduces angiogenesis process, and protects against lung and liver metastases in female BALBc mice. However, the active molecule from the Maitake Pro4X extract and which is the exact molecular mechanism to be utilized to act as tumor preventive agent are needed to be determined. Base upon of these results, we can postulated a Maitake D‐Fraction Pro4X as a good candidate to be used as preventive agents in breast carcinogenesis in a high‐risk population.

## Conflict of Interest

None declared.

## References

[1] Rini, B. I. , S. Halabi , J. E. Rosenberg , W. M. Stadler , D. A. Vaena , S. S. Ou , et al. 2008 Bevacizumab plus interferon alfa compared with interferon alfa monotherapy in patients with metastatic renal cell carcinoma: CALGB 90206. J. Clin. Oncol. 26:5422–5428. 18936475 10.1200/JCO.2008.16.9847 PMC2651074

[2] Larkin, J. , V. Chiarion‐Sileni , R. Gonzalez , J. Jacques Grob , C. Lance Cowey , et al. 2015 Combined Nivolumab and ipilimumab or monotherapy in untreated melanoma. N. Engl. J. Med. 373:23–34. 26027431 10.1056/NEJMoa1504030 PMC5698905

[3] Cummings, S. R. , J. A. Tice , S. Bauer , W. S. Browner , J. Cuzick , E. Ziv , V. Vogel , et al. 2009 Prevention of breast cancer in postmenopausal women: approaches to estimating and reducing risk. J. Natl Cancer Inst. 101:384–398. 19276457 10.1093/jnci/djp018 PMC2720698

[4] Białek, A. , A. Stawarska , A. Tokarz , K. Czuba , A. Konarska , and M. Mazurkiewicz . 2014 Enrichment of maternal diet with conjugated linoleic acids influences desaturases activity and fatty acids profile in livers and hepatic microsomes of the offspring with 7,12‐dimethylbenz[a]anthraceneinduced mammary tumors. Acta Pol. Pharm. 71:747–761. 25362803

[5] Illana‐Esteban, C . 2008 El hongo maitake (*Grifola frondosa*) y su potencial terapéutico. Rev. Iberoam Micol. 25:141–144. 18785781 10.1016/s1130-1406(08)70033-0

[6] Lull, C. , H. J. Wichers , and H. F. J. Savelkoul . 2005 Antiinflammatory and immunomodulating properties of fungal metabolites. Mediators Inflamm. 2:63–80. 16030389 10.1155/MI.2005.63 PMC1160565

[7] Kodama, N. , K. Komuta , and H. Nanba . 2002 Can maitake MD‐fraction aid cancer patients? Altern. Med. Rev. 7:236–239. 12126464

[8] Masuda, Y. , Y. Murata , M. Hayashi , and H. Nanba . 2008 Inhibitory effect of MD‐ Fraction on tumor metastasis: involvement of NK cell activation and suppression of intercellular adhesion molecule (ICAM)‐ 1 expression in lung vascular endothelial cells. Biol. Pharm. Bull. 31:1104–1108. 18520039 10.1248/bpb.31.1104

[9] Balogh, G. A. , D. J. Obiol , and E. N. Alonso . 2012 Maitake‐Fraction D y sus efectos terapéuticos en cáncer de mama. *in* EAE, Ed. AV Akademikerverlag GmbH & Co, Saarbrücken, Germany. ISBN: 978‐3‐659‐05372‐6.

[10] Masuda, Y. , T. Togo , S. Mizuno , M. Konishi , and H. Nanba . 2012 Soluble beta‐glucan from *Grifola frondosa* induces proliferation and Dectin‐1/Syk signaling in resident macrophages via the GM‐CSF autocrine pathway. J. Leukoc. Biol. 91:547–556. 22028332 10.1189/jlb.0711386

[11] Alonso, E. , M. Orozco , A. Nieto , and G. A. Balogh . 2013 Genomic signature induces by Maitake D‐fraction in breast cancer cells. J. Med. Food 16:602–617. 23875900 10.1089/jmf.2012.0222 PMC3719462

[12] AVMA . 2013 Guidelines for the euthanasia of animals, 2013 Edition. American Veteriary Medical Association ISBN 978‐1‐882691‐21‐0 Version 2013.0.1 Available at https://www.avma.org/KB/ Policies/Documents/euthanasia.pdf (accessed 9 September 2015).

[13] Sato, Y. , K. Mukai , S. Watanabe , M. Goto , and Y. Shimosaio . 1986 The AMeX method a simplified technique of tissue processing and parafin embedding with improved preservation of antigens for immunostaining. Am. J. Pathol. 125:431–435. 2432790 PMC1888473

[14] Lane, B. , and D. L. Europa . 1965 Differential staining of ultrathn sections of eponembedded tissues for light microscopy. J. Histochem. Cytochem. 13:579–582. 4159453 10.1177/13.7.579

[15] Costa, R. , R. Negrão , I. Valente , Â. Castela , D. Duarte , L. Guardão , et al. 2013 Xanthohumol modulates inflammation, oxidative stress, and angiogenesis in type 1 diabetic rat skin wound healing. J. Nat. Prod. 76:2047–2053. 24200239 10.1021/np4002898

[16] Alexander, B. , A. I. Fishman , M. Eshghi , M. Choudhury , and S. Konno . 2013 Induction of cell death in renal cell carcinoma with combination of D‐ fraction and vitamin C. Integr. Cancer Ther. 12:442–448. 23341484 10.1177/1534735412473643

[17] Sambrook, J. , E. F. Fritsch , and T. Maniatis . 1989 Molecular cloning: a laboratory manual. Cold Spring Harbor Laboratory Press, New York, NY.

[18] SAS Institute, Inc . 1996 SAS System 6.12. Cary, NC Statistical Analysis Systems Institute, Inc..

[19] Fullerton, S. A. , A. A. Samadi , D. G. Tortorelis , M. S. Choudhury , C. Mallouh , H. Tazaki , et al. 2000 Induction of apoptosis in human prostatic cancer cells with beta‐ glucan (Maitake mushroom polysaccharide). Mol. Urol. 4:7–13. 10851301

[20] Shomori, K. , M. Yamamoto , I. Arifuku , K. Teramachi , and H. Ito . 2009 Antitumor effects of a water‐ soluble extract from Maitake (Grifola frondosa) on human gastric cancer cell lines. Oncol. Rep. 22:615–620. 19639212 10.3892/or_00000480

[21] Soares, R. , M. Meireles , A. Rocha , A. Pirraco , D. Obiol , E. Alonso , et al. 2011 Maitake (D fraction) mushroom extract induces apoptosis in breast cancer cells by BAK‐ 1 gene activation. J. Med. Food 14:563–572. 21480800 10.1089/jmf.2010.0095

[22] Hishida, I. , H. Nanba , and H. Kuroda . 1988 Antitumor activity exhibited by orally administered extract from fruit body of *Grifola frondosa* (maitake). Chem. Pharm. Bull. (Tokyo) 36:1819–1827. 3203420 10.1248/cpb.36.1819

[23] Nanba, H. , and K. Kubo . 1997 Effect of Maitake D‐fraction on cancer prevention. Ann. N. Y. Acad. Sci. 833:204–207. 9616756 10.1111/j.1749-6632.1997.tb48611.x

[24] Nanba, H. 1995 Activity of maitake D‐fraction to inhibit carcinogenesis and metastasis. Ann. N. Y. Acad. Sci. 768:243–245. 8526356 10.1111/j.1749-6632.1995.tb12130.x

[25] Motamed, K. , and E. H. Sage . 1998 SPARC inhibits endothelial cell adhesion but not proliferation through a tyrosine phosphorylation‐dependent pathway. J. Cell. Biochem. 70:543–552. 9712151 10.1002/(sici)1097-4644(19980915)70:4<543::aid-jcb10>3.0.co;2-i

[26] Azim, H. A. Jr , S. Singhal , M. Ignatiadis , C. Desmedt , D. Fumagalli , I. Veys , et al. 2013 Association between SPARC mRNA expression, prognosis and response to neoadjuvant chemotherapy in early breast cancer: a pooled in‐ silico analysis. PLoS ONE 8:e62451. 23638089 10.1371/journal.pone.0062451 PMC3637211

[27] Hsiao, Y. H. , H. C. Lien , H. L. Hwa , W. H. Kuo , K. J. Chang , and F. J. Hsieh . 2010 SPARC (osteonectin) in breast tumors of different histologic types and its role in the outcome of invasive ductal carcinoma. Breast J. 16:305–308. 20210803 10.1111/j.1524-4741.2009.00899.x

[28] Witkiewicz, A. K. , B. Freydin , I. Chervoneva , M. Potoczek , W. Rizzo , H. Rui , et al. 2010 Stromal CD10 and SPARC expression in ductal carcinoma in situ (DCIS) patients predicts disease recurrence. Cancer Biol. Ther. 10:391–396. 20574156 10.4161/cbt.10.4.12449 PMC3040854

[29] Nagai, M. A. , R. Gerhard , J. H. Fregnani , S. Nonogaki , R. B. Rierger , M. M. Netto , et al. 2011 Prognostic value of NDRG1 and SPARC protein expression in breast cancer patients. Breast Cancer Res. Treat. 126:1–14. 20369286 10.1007/s10549-010-0867-2

[30] Koblinski, J. E. , B R. Kaplan‐Singer , S. J. Van Osdol , M. Wu , J. A. Engbring , S. Wang , et al. 2005 Endogenous osteonectin/SPARC/BM‐40 expression inhibits MDA‐MB‐231 breast cancer cell metastasis. Cancer Res. 65:7370–7377. 16103089 10.1158/0008-5472.CAN-05-0807

[31] Watkins, G. , A. Douglas‐Jones , R. Bryce , R. E. Mansel , and W. G. Jiang . 2005 Increased levels of SPARC (osteonectin) in human breast cancer tissues and its association with clinical outcomes. Prostaglandins Leukot. Essent. Fatty Acids 72:267–272. 15763438 10.1016/j.plefa.2004.12.003

[32] Lu, Y. , Y. Z. Lin , R. LaPushin , B. Cuevas , X. Fang , S. X. Yu , et al. 1999 The PTEN/MMAC1/TEP tumor suppressor gene decreases cell growth and induces apoptosis and anoikis in breast cancer cells. Oncogene 18:7034–7045. 10597304 10.1038/sj.onc.1203183

[33] Heikkinen, T. , D. Greco , L. M. Pelttari , J. Tommiska , P. Vahteristo , P. Heikkilä , et al. 2011 Variants on the promoter region of PTEN affect breast cancer progression and patient survival. Breast Cancer Res. 13:R130. 22171747 10.1186/bcr3076 PMC3326572

[34] Haagenson, K. K. , L. Tait , J. Wang , M. P. Shekhar , L. Polin , W. Chen , et al. 2012 Cullin‐ 3 protein expression levels correlate with breast cancer progression. Cancer Biol. Ther. 13:1042–1046. 22825334 10.4161/cbt.21046 PMC3461811

[35] Kossatz, U. , K. Breuhahn , B. Wolf , M. Hardtke‐Wolenski , L. Wilkens , D. Steinemann , et al. 2010 The cyclin E regulator cullin 3 prevents mouse hepatic progenitor cells from becoming tumor‐ initiating cells. J. Clin. Invest. 120:3820–3833. 20978349 10.1172/JCI41959 PMC2964969

[36] Kim, B. , H. J. Nam , K. E. Pyo , M. J. Jang , I. S. Kim , D. Kim , et al. 2011 Breast cancer metastasis suppressor 1 (BRMS1) is destabilized by the Cul3‐ SPOP E3 ubiquitin ligase complex. Biochem. Biophys. Res. Commun. 415:720–726. 22085717 10.1016/j.bbrc.2011.10.154

[37] Robey, R. W. , O. Polgar , J. Deeken , K. W. To , and S. E. Bates . 2007 ABCG2: determining its relevance in clinical drug resistance. Cancer Metastasis Rev. 26:39–57. 17323127 10.1007/s10555-007-9042-6

[38] Doyle, L. A. , W. Yang , L. V. Abruzzo , T. Krogmann , Y. Gao , A. K. Rishi , et al. 1998 A multidrug resistance transporter from human MCF‐ 7 breast cancer cells. Proc. Natl Acad. Sci. USA 95:15665–15670. 9861027 10.1073/pnas.95.26.15665 PMC28101

[39] Jiao, X. , L. Zhao , M. Ma , X. Bai , M. He , Y. Yan , et al. 2013 MiR‐ 181a enhances drug sensitivity in mitoxantoneresistant breast cancer cells by targeting breast cancer resistance protein (BCRP/ABCG2). Breast Cancer Res. Treat. 139:717–730. 23780685 10.1007/s10549-013-2607-x

[40] Ma, M. T. , M. He , Y. Wang , X. Y. Jiao , L. Zhao , X. F. Bai , et al. 2013 MiR‐ 487a resensitizes mitoxantrone (MX)‐ resistant breast cancer cells (MCF‐ 7/ MX) to MX by targeting breast cancer resistance protein (BCRP/ABCG2). Cancer Lett. 339:107–115. 23879965 10.1016/j.canlet.2013.07.016

[41] Butt, A. J. , K. A. Dickson , F. McDougall , and R. C. Baxter . 2003 Insulin‐ like growth factor‐ binding protein‐ 5 inhibits the growth of human breast cancer cells in vitro and in vivo. J. Biol. Chem. 278:29676–29685. 12777377 10.1074/jbc.M301965200

[42] Ahn, B. Y. , A. N. Elwi , B. Lee , D. L. Trinh , A. C. Klimowicz , A. Yau , et al. 2010 Genetic screen identifies insulin‐ like growth factor binding protein 5 as a modulator of tamoxifen resistance in breast cancer. Cancer Res. 70:3013–3019. 20354179 10.1158/0008-5472.CAN-09-3108

